# Shavenbaby Couples Patterning to Epidermal Cell Shape Control

**DOI:** 10.1371/journal.pbio.0040290

**Published:** 2006-08-22

**Authors:** Hélène Chanut-Delalande, Isabelle Fernandes, Fernando Roch, François Payre, Serge Plaza

**Affiliations:** Centre de Biologie du Développement, CNRS UMR 5547, Université Paul Sabatier, Toulouse, France; Cambridge University, United Kingdom

## Abstract

It is well established that developmental programs act during embryogenesis to determine animal morphogenesis. How these developmental cues produce specific cell shape during morphogenesis, however, has remained elusive. We addressed this question by studying the morphological differentiation of the *Drosophila* epidermis, governed by a well-known circuit of regulators leading to a stereotyped pattern of smooth cells and cells forming actin-rich extensions (trichomes). It was shown that the transcription factor Shavenbaby plays a pivotal role in the formation of trichomes and underlies all examined cases of the evolutionary diversification of their pattern. To gain insight into the mechanisms of morphological differentiation, we sought to identify *shavenbaby*'s downstream targets. We show here that Shavenbaby controls epidermal cell shape, through the transcriptional activation of different classes of cellular effectors, directly contributing to the organization of actin filaments, regulation of the extracellular matrix, and modification of the cuticle. Individual inactivation of *shavenbaby*'s targets produces distinct trichome defects and only their simultaneous inactivation prevent trichome formation. Our data show that *shavenbaby* governs an evolutionarily conserved developmental module consisting of a set of genes collectively responsible for trichome formation, shedding new light on molecular mechanisms acting during morphogenesis and the way they can influence evolution of animal forms.

## Abstract 2

The authors explore how Shavenbaby (Svb) orchestrates the formation of specialized actin-rich structures, called trichomes, that underlie the distinctive morphology of dorsal hairs and ventral denticles.

## Introduction

A general feature of development is the control of tissue and cell morphogenesis, a process whereby each cell acquires a specific shape depending upon its individual identity. Although the mechanisms that permit the patterning of a cellular field are now relatively well understood in different systems, how cell fate becomes translated into cell-shape control remains largely unknown.

Genetic analysis of epidermal differentiation in *Drosophila* has made this process a paradigm for our attempts to understand the subsequent steps of morphogenesis in animals. During early embryogenesis, cascades of transcription factors progressively refine the different cellular territories and define their respective identity [1]. Signaling pathways then act to establish boundaries between adjacent cell fields (reviewed in [2,3]) and participate in the regulation of the size of these territories through the control of cell survival [4]. Finally, when the epidermis is composed of a monolayer of post-mitotic cells, signaling pathways specify cell fates, ultimately determining morphological differentiation [1,2,5]. Epidermal cells in a given embryonic segment display a stereotyped pattern of two distinct morphological fates: cells with a smooth apical surface, which generate naked cuticle, and cells producing actin-rich cytoplasmic extensions, which eventually become the mature larval microtrichiae or trichomes, generally called denticles (ventral) and hairs (dorsal).

**Figure 1:**
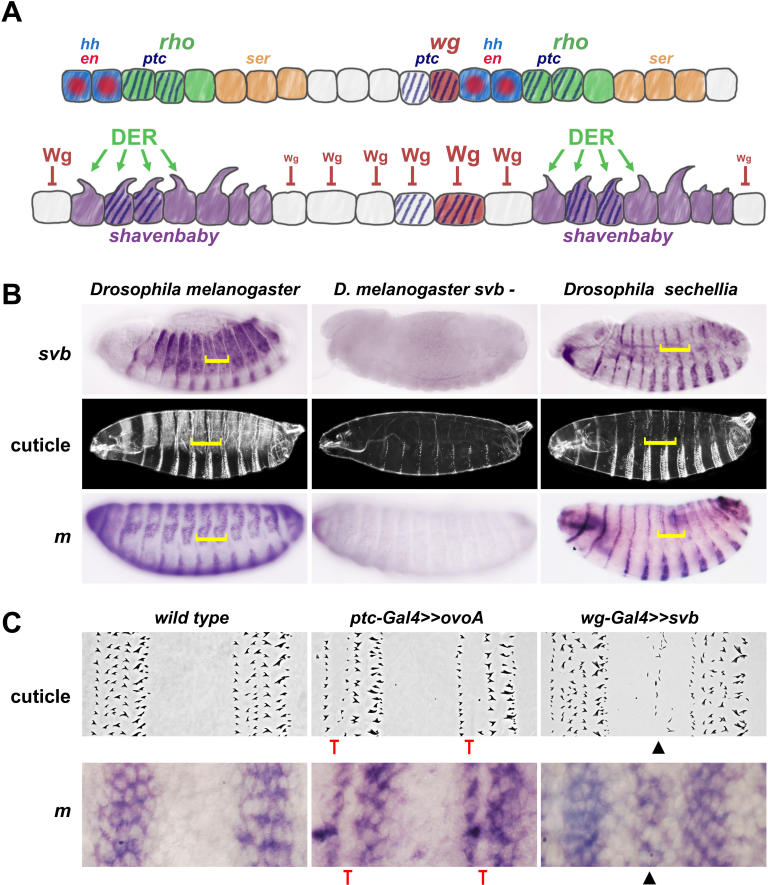
Control of *m* Expression by *svb* in the Embryonic Epidermis (A) Schematic representation of the signaling pathways that control morphological differentiation of the ventral embryonic epidermis, at stage 12 (top) and at the end of embryogenesis (bottom); anterior is to the left. In addition to *engrailed (en),* posterior cells express *Hedgehog (Hh),* and *patched (ptc)* is expressed in a two-cell-wide stripe on each side of the *Hh*-expressing cells*. Hh,* together with *serrate (Ser),* activates *rhomboid (Rho)* expression in a three-cell wide stripe. The Rhomboid protease activates the ligand of the d-EGF receptor (DER), whose activation triggers the expression of *svb,* resulting in the formation of six to seven rows of denticles. Wingless, which is expressed in the posterior-most row of anterior cells, diffuses asymmetrically and represses *shavenbaby* transcription to form naked cuticle. (B) Whole-mount in situ hybridization of *svb* (top) and *m* (bottom) mRNA; cuticles are shown in the middle panels. Inactivation of *svb* prevents the formation of most trichomes and abolishes *m* epidermal expression. *m* expression foreshadows the pattern of trichomes in D. melanogaster and *D. sechellia* larvae. Yellow brackets outline two dorsal segments. (C) Close-up of the cuticle region corresponding to the third (A3) and fourth (A4) abdominal segments (top) and *m* mRNA distribution (bottom), in wild-type D. melanogaster embryos (left), ptc-Gal4-driven expression of UAS-OvoA (middle) and expression of UAS-*svb* under the control of wg-Gal4 (right). Whereas in wild type, *m* is expressed in each segment in a five to seven–cell-wide stripe, the expression of OvoA prevents the formation of denticle rows 2–3 and represses *m* expression in the corresponding cells (red lines). Reciprocally, ectopic expression of *svb* in *wg* cells triggers the formation of supernumerary denticles and ectopic expression of *m* (arrowheads).

In the ventral region of the abdomen, each segment possesses six to seven rows of pigmented denticles, which are involved in locomotion. Activation of the Wingless (Wg) pathway determines the naked fate [6], whereas activation of the d-EGF-receptor (DER) pathway promotes denticle formation [7]. In addition, Serrate-Notch signaling also contributes to define the denticle field [8,9] through a fine-tuning of the extent of DER activation [10]. The activities of Wg and DER pathways on epidermal differentiation converge in the transcriptional regulation of *shavenbaby (svb)* [11]. DER activates *svb* transcription in denticle-forming cells, whereas Wg acts as a repressor in the naked territories (Figure 1A), leading to the precise expression of *svb* in denticle cells. In turn, *svb* is required for denticle formation, since *svb* mutants display essentially naked cuticle. In addition, the ectopic expression of *svb* in smooth cells is sufficient to produce cuticular extensions [11], which nevertheless do not display the typical morphology of denticles, likely because those cells lack auxiliary factors involved in the fine shaping of these structures. *svb,* which encodes a zinc finger transcription factor [12–14], is the most-downstream regulator so far known to specify the denticle pattern [11].

Additional evidence supports the hypothesis that, whereas all other upstream regulators act to define epidermal cell fates, *svb* controls epidermal cell shape changes. Although different mechanisms specify morphological fates in the dorsal epidermis [15], *svb* is required for the formation of dorsal hairs as it is for denticles. It has been shown that the modification of *svb,* leading to the restriction of its expression in a subset of dorsal epidermal cells, is the unique cause of the modification of the trichome pattern in a sibling Drosophila melanogaster species, *D. sechellia,* in which several rows of dorsal hairs are replaced by naked cuticle [16]. Somewhat surprisingly, given the large number of genes that are theoretically capable of modifying dorsal hair patterning [1], all cases of dorsal hair–pattern evolution examined in dipterans are the consequence of the modification of *svb* expression only [16–18]. Thus, developmental and evolutionary studies show that *svb* is critical for epidermal differentiation and that the presence/absence of *svb* expression ultimately determines the pattern of denticles and dorsal hairs [19].

How does *svb* control the formation of denticles and dorsal hairs during epidermal differentiation? Little is known about the cellular mechanisms underlying trichome formation. Epidermal cells are highly polarized along the apico-basal axis, with actin microfilaments accumulating at the cell cortex where they interact with apical junction complexes [20,21]. The formation of denticles and dorsal hairs is initiated by F-actin accumulation and reorganization, leading to the production of an apical bundle in the posterior region of the cell [14,22,23]. Each bundle then grows perpendicularly to the apical cell surface [22,23] and probably provides the mechanical force for the modification of the apical membrane. At later stages, ecdysteroid hormones induce epidermal cells to synthesize components of the cuticular envelopes, which are modified by the catecholamine biosynthetic pathway to ensure trichome pigmentation and hardening [24]. Despite the large number of genetic screens based upon cuticle observation [25,26], the molecular mechanisms responsible for the different steps of epidermal differentiation still remain to be uncovered.

Since *svb* activity acts cell autonomously to promote denticle formation [11,14], we searched for genes whose expression is controlled by *svb* as an alternative approach to identify players responsible for the morphological differentiation of epidermal cells. We identified several cellular factors involved directly in subsequent steps of denticle formation. Although actin remodeling is generally regarded as the outcome of post-translational modifications, we show that denticle formation relies on the *svb*-dependent transcriptional activation of several factors involved in microfilament formation or bundling. We provide evidence that *svb* directly controls the transcription of *miniature (m),* which encodes a membrane protein containing a Zona Pellucida (ZP) domain, and we show that *m* is required for the membrane/cuticle interaction in the denticle. We also identified a *svb* target responsible for denticle pigmentation, therefore demonstrating that *svb* coordinates several aspects of denticle differentiation. Finally we show that *svb* regulates the same set of genes for the formation of dorsal hairs and suggest that the evolution of the dorsal hairs pattern in D. sechellia results from the concerted modification of the expression of cellular effectors regulated by *svb*.

## Results

### *svb* Directs *m* Expression in the Embryonic Epidermis

As a first step towards identifying players involved in denticle formation, we searched for genes expressed in epidermal cells by conducting a systematic survey of expression patterns available from the Berkeley Drosophila Genome Project [27] and the literature (Table S1). Among the approximately 400 genes reported to be transcribed in the epidermis, we found that only a small number (<10%) are expressed in a segmentally repeated pattern, at a time of epidermal cells morphological differentiation. We further analyzed the epidermal expression of these genes by in situ hybridization and tested for its dependence on *svb* (Table S1). We first focused on *m* [28], because its expression in stage-15 embryos strikingly resembles the trichome pattern, which is specified by *svb* at previous stages (Figure 1B).

We found that *m* expression in epidermal cells is abolished in *svb* mutant embryos (Figure1B), showing that *svb* is required for *m* transcription in the epidermis. Detailed examination (Figure 1C) revealed a precise correlation between *m* expression and the denticle pattern in wild-type embryos, with a six to seven–cell-wide expression stripe in each segment that alternates with a band of *m*-negative cells. OvoA is a germinal isoform of the *ovo*/*svb* gene that acts as a transcriptional repressor [13], able to counteract *svb* activity when its expression is artificially directed in the epidermis [13,14]. When expressed in *ptc*-expressing cells (see Figure 1A), OvoA leads to the replacement of denticle rows 2 and 3 by a stripe of naked cuticle interrupting each denticle belt (Figure 1C). This OvoA expression also represses *m* transcription in the epidermal cells corresponding to denticle rows 2 and 3, providing evidence that *svb* activity is required cell autonomously for *m* expression in denticle cells. To test whether *svb* is sufficient to induce *m* expression in the epidermis, we examined the consequence of the ectopic expression of *svb* in smooth cells. Ectopic expression of *svb* in *wg*-expressing cells (Figure 1A) causes the formation of a supernumerary row of cuticular extensions and leads to an ectopic stripe of *m* expression (Figure 1C), showing that *svb* is sufficient to trigger *m* transcription in epidermal cells.

As the evolutionary loss of dorsal trichomes in D. sechellia embryos is the result of the restriction of *svb* expression to narrow stripes in dorsal epidermal cells [16], we asked whether *m* expression was modified accordingly in D. sechellia. When compared to *D. melanogaster, m* expression in D. sechellia embryos is indeed seen to be restricted to narrow bands in the dorsal region. These data thus bring independent support to the conclusion that *m* expression is controlled by *svb* in the embryonic epidermis and show that this regulation has been evolutionarily conserved.

All together, our results demonstrate that *svb* activity is necessary and sufficient to control *m* expression specifically in epidermal cells producing denticle and dorsal hairs.

### Shavenbaby Directly Controls Miniature Transcription

To investigate whether *svb* directly regulates *m* expression, we first attempted to identify *cis*-regulatory elements directing *m* transcription in the embryonic epidermis.

**Figure 2:**
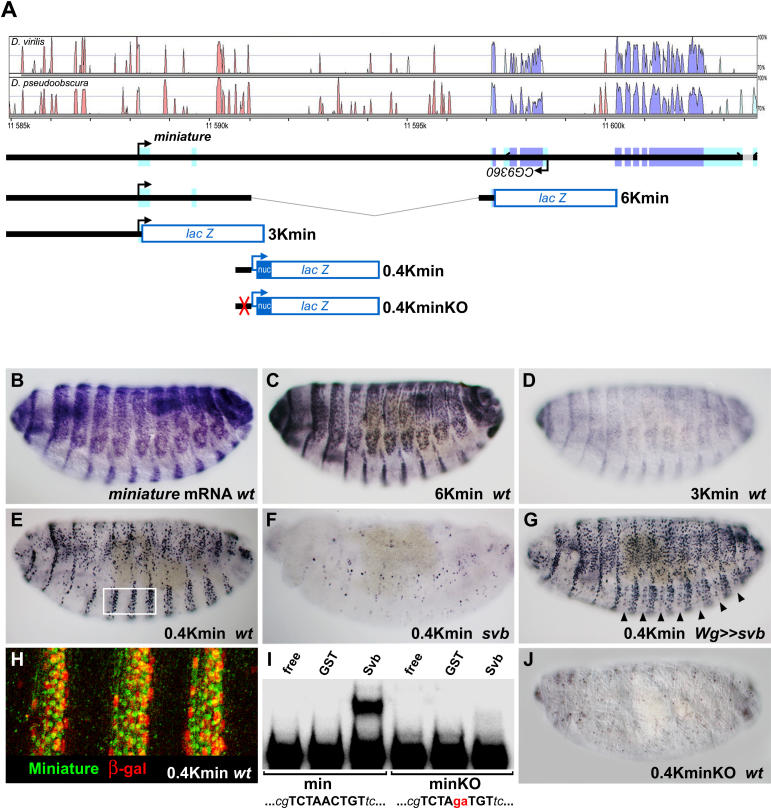
An Evolutionarily Conserved Enhancer Directs the *svb*-Dependent Expression of *m* (A) Evolutionary conservation of the *m* locus and summary of transgenic reporter constructs. Transcribed regions of *m* span more than 15kb and harbor an unrelated gene, *CG9360,* transcribed from the complementary stand. Histograms plot the level of sequence conservation between D. melanogaster and D. pseudoobscura (top) or D. virilis (bottom), as represented from the Vista Genome Browser package. Red, light blue, and dark blue peaks correspond to conserved sequences in non-coding regions, 3′ and 5′ UTR, and translated sequences, respectively. Genomic regions displaying high evolutionary conservation were fused with reporter LacZ genes, encoding either a cytoplasmic (6Kmin and 3Kmin) or nuclear (0.4Kmin) β-gal enzyme, and used to generate transgenic lines. (B and C) Compared to *m* mRNA (B), 6Kmin constructs reproduce endogenous *m* expression (C). (D) Deletion of intronic sequences leads to a strong reduction of staining in 3Kmin constructs. (E) The 0.4Kmin construct drives *m*-like epidermal expression at a high level. The white box indicates the ventral region selected for the close-up presented in panel (H). (F and G) Like the endogenous gene, this enhancer is responsive to *svb,* since staining is absent in *svb* mutants (F) and additional stripes are produced after *svb* ectopic expression (arrowheads) (G). (H) Close up of the ventral region (segments A3–A5) of a 0.4Kmin embryo, showing that the β-gal reporter (red) is co-expressed with endogenous Miniature protein (green) in epidermal cells. (I and J) Electrophoresis mobility shift assays show the specific binding of the Svb protein to wild-type *m* enhancer. Introduction of 2 point mutations prevents in vitro binding (I) and leads to an inactive enhancer when assayed in vivo (J). Sequence of the Svb binding site (capital letters) and introduced mutations (red) are indicated.

We generated transgenic lines in which a reporter gene is placed under the control of a 6-kilobase (kb) genomic region encompassing the *m* transcriptional start site (6Kmin) (see Figure 2A). These 6Kmin reporter transgenes faithfully reproduce *m* expression in late embryos (Figure 2B and 2C) and display a similar dependence on *svb* activity (unpublished data). Deletion of most intronic sequences to a 3-kb fragment leads to a strong diminution of the expression (Figure 2D), showing that *cis*-regulatory elements required for *m* epidermal expression are located in intronic regions. We focused on a 0.4-kb region from the second *m* intron, which is highly conserved in Drosophilidae (Figure 2A). This fragment, when placed upstream of a minimal promoter, promotes a robust expression in the epidermis (Figure 2E), similar to that of the endogenous *m* gene (Figure 2B–2H). Expression of 0.4Kmin lines is lost in a *svb* mutant background (Figure 2F), whereas an additional stripe of expression appears when *svb* is ectopically expressed in smooth cells (Figure 2G). Therefore, this 0.4-kb region of the *m* gene behaves as a *svb*-responsive element, able to drive epidermal expression under the control of *svb*.

We then assayed the ability of the Svb protein to bind in vitro to the corresponding DNA region. Gel retardation assays showed that the recombinant Svb protein binds specifically to the 0.4-kb *m* element through a single region, further refined to a 200-base pair (bp) fragment (Figure 2I). Sequence analysis revealed the presence of a nucleotide motif matching (7/7) the consensus binding site previously defined for Ovo proteins [29], which share the same DNA binding domain with Svb [12]. Additional DNA-binding experiments showed that Svb binds to this predicted site with a high affinity (estimated Kd = 2.5 ± −0.8 nM, unpublished data). The sequence of this Svb binding site on the *m* enhancer (at position chrX:11487686, on the genomic scaffold) is conserved in all sequenced *Drosophila* species. Introduction of point mutations that substitute two invariant nucleotides found in all Ovo binding sites [29] eliminates Svb binding in vitro (Figure 2I), showing that Svb interacts specifically with the *m* enhancer through this single binding-site. To test the consequence of preventing Svb binding in vivo, we introduced these two mutations into the 0.4-kb enhancer construct and generated corresponding transgenic lines (0.4KminKO). All the tested 0.4KminKO lines (12) lost *m*-like epidermal expression, with only some variable residual staining, variable from line to line (Figure 2J).

These results demonstrate that Svb binds to an evolutionarily conserved element of the *m* gene, which behaves as a *svb*-responsive enhancer in the embryonic epidermis. Mutations preventing Shavenbaby binding to this element abolish its enhancer activity, thus providing strong evidence that Svb exerts a direct control on *m* transcription.

### Miniature Is Required for Denticle Formation

*m* encodes a membrane protein [30], which has recently been implicated in actin-based remodeling of wing cell shape during metamorphosis [28,31]. To examine whether *m* also plays a role in embryonic epidermal cells, we analyzed consequences of *m* inactivation on denticles.

**Figure 3:**
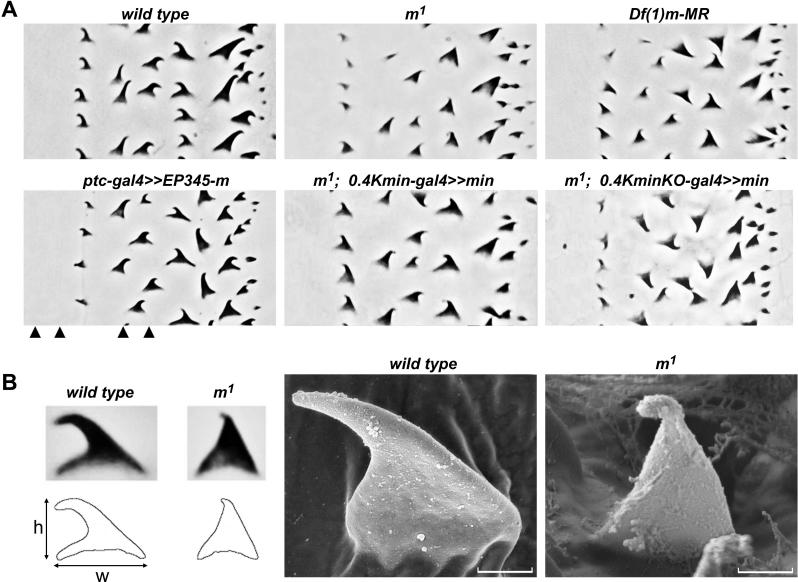
*m* Impinges on Denticle Formation (A) Consequences of alterations of *m* function and expression on denticle formation. The *m^1^* mutation leads to morphological alteration of denticles, with a characteristic abnormal triangular shape. A similar phenotype is observed with *Df(1)m-MR,* a deficiency in which the entire *m* locus is deleted. Overexpression of *m,* by crossing the Ptc-Gal4 driver line to EP345-m, does not modify the cuticular phenotype in the corresponding regions (arrowheads). Re-expression of wild-type *m* products driven by the 0.4Kmin-gal4 transgenic lines, but not 0.4KminKO-Gal4 lines, rescues the characteristic denticles defects of *m^1^* embryos. All pictures correspond to the A4 segment. (B) High magnification views of wild-type *(wt)* and *m^1^* denticles (fourth rows of A4), as observed in either light (left) or scanning electron microscopy (right). Whereas the absence of *m* does not affect the denticle height (h), the width (w) is reduced, producing denticles of smaller area that display an abnormal shape lacking the median constriction characteristic of wild-type denticles. See Table S2 for quantification of these defects.

Although the absence of *m* does not affect the denticle pattern, detailed examination shows that denticles are altered in *m* mutant embryos (Figure 3). In wild type, each denticle row has a characteristic morphology, but all denticles share a common organization resembling a rose thorn: a large base, followed by a median constriction forming a narrow neck, which supports the distal hook, which points either anteriorly or posteriorly (Figure 3). In larvae carrying the *m^1^* loss-of-function mutation, or the *Df(1)m-MR* deficiency that deletes the entire *m* gene [28], denticles are small and misshapen (Figure 3 and Table S2); the median constriction is absent, leading to poorly differentiated denticles that display an aberrant triangular shape. These mutant defects are rescued when *m* is re-expressed using the wild-type 0.4Kmin element as a GAL4 driver (Figure 3A). These results provide additional evidence that this *m* enhancer is fully functional in the epidermis and demonstrate that the observed denticle defects are due to the loss of *m* activity.

To further investigate the role of *m* in denticle formation, we tested the influence of its ectopic expression in wild-type embryos. We used both an EP (target P-element) insertion located 2 kb upstream of the *m* start site, or the min cDNA placed under the control of UAS sequences (Gal4 binding sites), which both allow a strong *m* ectopic expression upon crossing with Gal4 drivers (unpublished data). None of the drivers we tested led to cuticular defects. For example, overexpression of *m* with the *ptc-gal4* driver, both in denticle rows 2 and 3 and in a two-cell-wide stripe in naked cells (see Figure 1), does not affect cuticle organization (Figure 3A). Therefore, *m* expression is required, but not sufficient, for the correct formation of denticles.

These results show that *m* is directly involved in a specific aspect of denticle formation. All together, these data further demonstrate that *svb* directly impinges on epidermal cell morphogenesis through the direct control of *m* transcription.

### Mutations Altering Denticles Allow the Identification of Additional *svb* Targets

The inactivation of *m* is not sufficient to mimic the *svb* phenotype, indicating that *svb* must regulate additional targets to achieve denticle formation. Mutations known so far to prevent denticle formation affect either *svb* or upstream regulators, therefore suggesting that inactivation of a given *svb* downstream target produces denticle malformation rather than prevents denticle formation. To test this hypothesis, we searched extensively for genes affecting denticle morphology (Table S1) and examined whether they are regulated by *svb*.

A dozen genes reported to affect denticle formation lead, when inactivated, to general cuticle defects, which are not, however, specific to trichomes (Table S1 and references therein). These genes (e.g., the Halloween family that encodes members of the ecdysteroid pathway) are either not expressed in the epidermis (e.g., are restricted to the ring gland) or display ubiquitous expression in epidermal cells (Table S1 and references therein).

**Figure 4:**
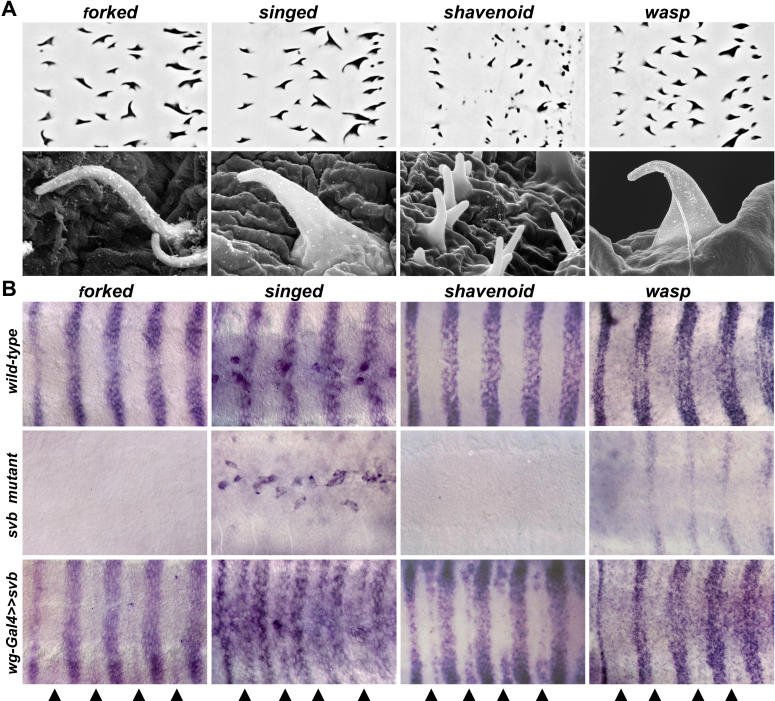
*svb* Directs the Expression of Genes Encoding Actin-Remodeling Proteins Required for Denticle Formation (A) Cuticle preparations showing denticle morphology in *f^36a^ sn^3^ sha^1^* and *wsp^3^* mutants. All views correspond to the same region, i.e., the ventral-most region of the A4 segment. Close-ups are scanning electron microscopy magnification of a representative denticle from the fourth row of denticles. (B) mRNA expression of *forked, singed, shavenoid/kojak,* and *wasp* in the ventral embryonic abdomen (A2–A6) of wild-type, *svb* mutants, and embryos expressing *svb* in *wg* cells, as observed from in situ hybridization. Arrowheads point to *wg* cells. Anterior is to the left in all pictures.

Mutations corresponding to seven genes affect denticles, without altering either the general cuticle organization or embryonic patterning (Table S1). While the expression of *fritz, hem, tricornered,* and *Stubble* are ubiquitous or undetectable in the embryonic epidermis, *forked (f), singed (sn),* and *shavenoid/kojak (sha)* display a segmental pattern of expression (Table S1). Mutations in both *sn* and *f* lead to similar defects [22], with thin, crooked abnormal denticles, whereas *sha* mutants display a distinct phenotype [25,32] with small, misshapen, multiply split denticles (Figure 4A and Table S2). Detailed analysis of mRNA distribution revealed that *sn, f,* and *sha* are specifically expressed in denticle cells (Figure 4B). The epidermal expression of their transcript is strongly reduced in *svb* mutants (Figure 4B), revealing that *svb* is necessary for *sn, f,* and *sha* expression in denticle cells. In addition, *svb* is sufficient to promote *sn* and *sha* expression in the epidermis, since their mRNA is robustly expressed in additional stripes following ectopic expression of *svb* (Figure 4B). The *svb*-driven ectopic expression of *f* is barely detectable, indicating that additional factors are required to promote *f* expression in naked cells. Taken together, these data show that the transcription of genes previously implicated in denticle formation is specifically activated by *svb*.

*sn, f,* and *sha* encode actin-associated factors [32,33], and a recent study has shown that other components of the actin cytoskeleton (Diaphanous, Enabled, and the members of Arp2/3 complex) accumulate in forming denticles [23]. This prompted us to analyze the embryonic expression of these genes, as well as additional actors in actin remodeling *(chickadee, gelsolin,* and *twinstar)*. All exhibit ubiquitous expression in embryos, which is not influenced by *svb* activity (Table S1). However, we found that *wasp (wsp)*, which encodes an activator of the Arp2/3 actin nucleation complex [34], is specifically expressed in denticle cells, in a *svb*-dependent manner (Figure 4B). The epidermal expression of *wsp* is strongly reduced in *svb* mutants and ectopic expression of *svb* leads to additional bands of *wsp* mRNA in naked cells, albeit at a much weaker level than in denticle cells (Figure 4B). Cuticle analysis revealed that zygotic *wsp* mutations [35] lead to the alteration of denticles, which are abnormally thin and often display bent extremities (Figure 4A). These defects are likely due to the lack of activation of the Arp2/3 complex, since the zygotic inactivation of two ubiquitous Arp2/3 subunits (ArcP34 and ArcP41) causes denticle alterations similar to those observed in *wsp* mutants (unpublished data).

Consistent with the idea that actin reorganization is required for denticle formation, our results show that Svb activates the expression of a subset of genes controlling actin dynamics, whose individual inactivation produces defects in denticle morphology.

### *svb*-Regulated Genes Functionally Collaborate in Denticle Formation

Inactivation of each *svb* target we identified leads to specific defects in the morphology of denticles, but is not sufficient to inhibit their formation. This suggests either that these genes are involved in nonessential aspects of denticle formation (e.g., fine shaping) or, alternatively, that denticle formation relies on the collective activity of numerous effectors, none of them being absolutely indispensable. To discriminate between these possibilities, we analyzed embryos carrying combinations of mutations in different *svb* targets.

**Figure 5:**
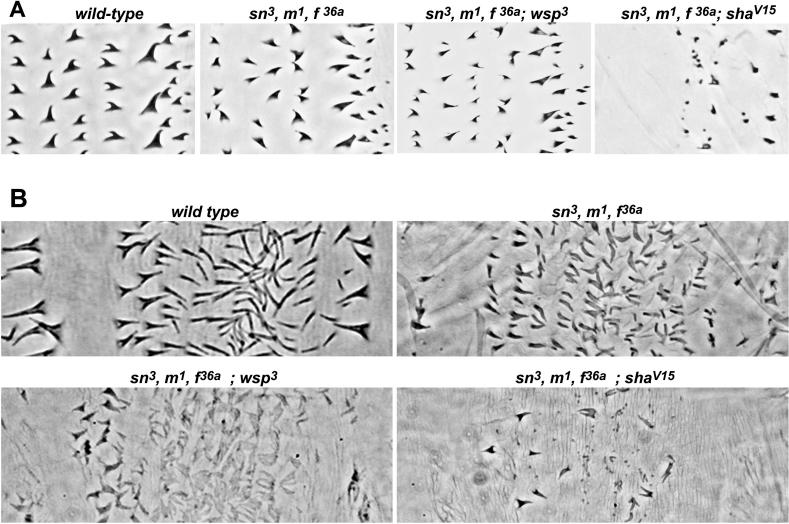
*svb* Downstream Targets Act Collectively in the Formation of Both Denticle and Dorsal Hairs (A) Denticle defects resulting from the combinations of individual mutations of *sn^3^, f^36a^, m^1^, sha^1^,* and *wsp^3^* (views of the ventral region of the A4 segment). Embryos triple mutant for *m, sn,* and *f* display an aggravated phenotype with respect to each simple mutant or double mutant. This leads to poorly differentiated denticles, which display an extremely thin tip and a small triangular base. In addition, the lateral spacing of mutant denticles is reduced, a consequence of denticle splitting with two tiny extensions side by side. The combined inactivation of *sn, f, m,* and *wsp* further increases the severity of mutant phenotypes, producing very small and highly abnormal denticles. Similarly, in embryos simultaneously lacking *sn, f, m,* and *sha,* the few remaining extensions are atrophic, and numerous denticles are replaced by naked cuticle. (B) *svb* downstream targets are required for dorsal hair formation. The dorsal region of a wild-type abdominal segment displays a stereotyped arrangement of epidermal extensions presenting a specific morphology: a row of large trichomes pointing anteriorly, a stripe of naked cuticle, three rows of extensions of intermediate size, and several rows of thin hairs. The cumulated inactivation of *svb* targets profoundly impairs dorsal hair formation. Hairs that display the superimposition of single mutant phenotypes are thickened *(m)*, crooked *(sn, f)* and split *(f, wsp, sha)*. In *sn^3^, f^36a^, m^1^* triple mutants, dorsal hairs are severely reduced in size and, in several cases, the formation of dorsal hair is abrogated, leaving abnormal naked regions, as best seen in the first row of trichomes. These phenotypes are even more pronounced following the combination with *wsp* mutation, and culminate in embryos lacking *m, sn, f,* and *sha,* where most dorsal hairs are absent and replaced by naked cuticle. In some cases, atrophic dorsal hairs are seen as duplicated spots, revealing hair splitting as observed in *sha* and *f* embryos. All pictures correspond to the A4 segment.

The phenotype of *sn, f* double mutants is slightly aggravated when compared to that of *sn* or *f* mutants (Table S2). When *m* mutation is combined with either *sn* or *f,* denticles display both the crooked extremities found in *sn* and *f,* and the defects characteristic of *m*. In addition, the inactivation of *m* and *f* (or *sn*) causes denticle splitting, as revealed by a significant increase in the number of abnormal denticles per row (Table S2). Simultaneous inactivation of all three genes leads to small and highly misshapen denticles that are more affected than in double mutants (Figure 5A and Table S2). Adding a fourth mutation, *wsp* or *sha,* further impairs the formation of denticles, leading in many cases to their replacement by naked regions (Figure 5A). The cumulated inactivation of *svb* targets thus progressively leads to tiny and highly malformed denticles, ending in the inhibition of denticle formation (Figure 5A). A similar effect is observed in the dorsal region, where combining mutations in *svb* targets ultimately prevents dorsal hair formation, in addition to producing cumulative morphological defects (Figure 5B). These results demonstrate that the morphological differentiation of the embryonic epidermis requires the concerted action of multiple genes, all playing a direct role in the formation both of denticles and dorsal hairs.

**Figure 6:**
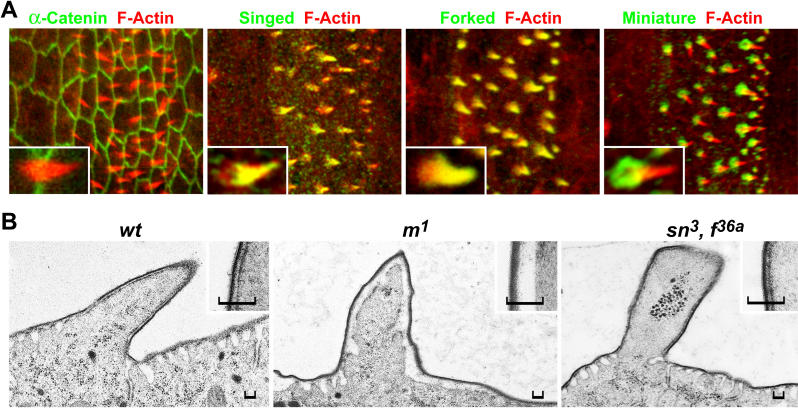
*svb* Target Genes Are Involved in Separate Features of Denticle Edification (A) Subcellular localization of Singed, Forked, and Miniature in the epidermis of stage 15 wild-type embryos. Distribution of α-catenin, a component of adherens junctions that underlines the cell contour, was observed in embryos expressing α-catenin-GFP driven by E22C-Gal4. Red indicates F-actin, and green indicates α-catenin, Singed, Forked, and Miniature. (B) Transmission electron microscopy analysis of denticle cells from wild-type *(wt)*, *m^1^,* and *sn^3^, f^36a^* double mutant embryos. As in smooth cells, the flat region of the apical cell face organizes microvilli that contact cuticle layers only at the apex. In contrast, the plasma membrane is in close contact with cuticle along the entire wild-type denticle contour. Although the *m^1^* mutation alters this membrane/cuticle contact, no defects are visible in *sn^3^, f^36a^* embryos. Close-up pictures show a region of the growing extension. Scale bar represents 0.25 μm.

To better understand the role of *svb* targets, we analyzed the subcellular distribution of Singed, Forked, and Miniature proteins at the onset of denticle formation. Forked, Singed, and Miniature are sequentially accumulated in denticle cells (Figure S1) and, consistent with their bundling activity, both Singed and Forked co-localize with F-actin in growing denticles (Figure 6A). Forked accumulates in nascent denticles, from the earliest steps of F-actin reorganization, whereas Singed appears slightly later, when the apical bundle is already formed and is continuing to grow. Miniature appears concomitantly with Singed and is initially detected in the cytoplasm of denticle cells (Figure S1). In later embryos (stage 15 and 16), Miniature accumulates in a restricted apical domain that underlines the base of each denticle (Figure 6A). Since Miniature is a membrane protein, this observation suggests the existence of a denticle-specific membrane subdomain. We therefore analyzed mutant defects at the ultrastructural level. In stage 16 wild-type embryos, the apical surface of epidermal cells organizes a dense array of microvilli that contact the cuticle only at their distal tips (Figure 6B). In contrast, there is a continuous and tight interaction between plasma membrane and cuticle layers in the denticle region (Figure 6B). Although naked cells are unaffected, denticle cells do not contact cuticle correctly in *m* mutant embryos (Figure 6B). The apical membrane is disorganized, with an abnormal gap between the denticle membrane and cuticle layers, indicating that *m* is required for the membrane/cuticle interaction that is specific to denticle cells. These defects are not observed in *sn, f* mutants (Figure 6B), confirming that they are specific to the lack of *m* activity.

Therefore, these data reveal that denticle formation requires, besides actin remodeling, a specific modification of the membrane/cuticle interaction that is directly dependent on the localized activity of Miniature. All together, these results show that *svb* directs the expression of cellular effectors involved in different steps and aspects of cell shape control, which are collectively responsible for denticle formation.

### *svb* Controls Denticle Pigmentation in the Embryonic Epidermis through the Regulation of *yellow* Transcription

**Figure 7:**
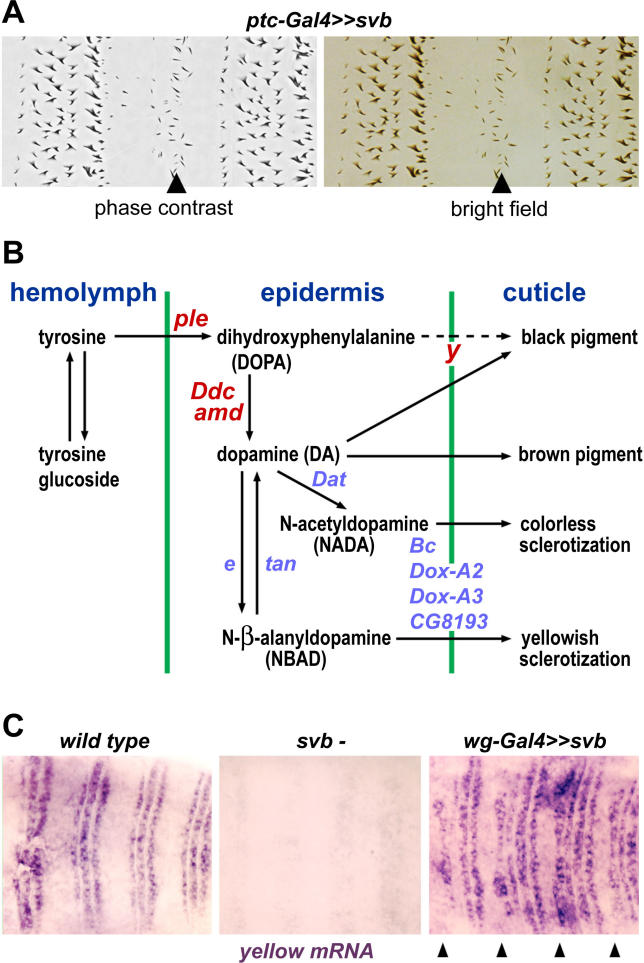
*svb* Controls Denticle Pigmentation through the Regulation of *y* Transcription (A) Cuticle preparations (A3–A4 segments) showing the effect of Ptc-Gal4–driven ectopic expression of *svb*. Ectopic cuticular extensions (arrowheads) are as densely pigmented as normal denticles, as demonstrated by bright-field observation (right). (B) Summary of the cuticular pigmentation pathway, showing genes that are putatively implicated in the transformation of tyrosine to pigmented compounds. Of the ten genes thought to be involved in pigment production we tested, only four (red) are expressed in the embryonic epidermis at the time of cuticle formation. (C) In situ hybridization showing the expression of *y* mRNA in the epidermis in late embryos. In wild type, *y* mRNA accumulates in three stripes per segment. Due to deep morphological folds at this stage, it is hard to define accurately the number of cell rows that express *y* in each segment. *y* transcription is controlled by *svb,* since staining is strongly decreased in *svb* mutant and an ectopic stripe of *y* mRNA (arrowheads) results from *svb* ectopic expression in *wg* cells.

In addition to the reorganization of cell shape, denticle formation implies pigmentation of cuticle extensions. We noticed that the supernumerary cuticular extensions formed after ectopic expression of *svb* are as darkly pigmented as normal denticles (Figure 7A), thus suggesting that *svb* also controls the expression of pigmentation genes in the ventral epidermis.

Cuticle pigmentation results from the activity of the catecholamine biosynthetic pathway, which transforms tyrosine into DOPA derivatives that are responsible for both sclerotization and coloration of the cuticle (reviewed in [24]). We examined the embryonic expression of ten genes putatively involved in pigmentation [24]; among them, four are expressed in the epidermis: *pale (ple), dopadecarboxylase (Ddc), alpha-methotrexate deficient (amd),* and *yellow (y)* (Figure 7B). While the expression of *ple, Ddc,* and *amd* are unaffected in *svb* mutants, we found that *y* is regulated by *svb*. Although *y* expression is strongly up-regulated in a subset of denticle cells in late wild-type embryos , this expression is undetectable in *svb* mutants (Figure 7C). Furthermore, ectopic expression of *svb* turns on *y* transcription in additional cell stripes (Figure 7C), showing that *svb* regulates *y* expression in epidermal cells.

Therefore, in addition to cytoskeletal organization, *svb* also controls cuticle pigmentation through the up-regulation of *y* expression in denticle cells.

## Discussion

Using denticle pattern as a readout of developmental cues, generations of fly geneticists have collectively gained a rare insight into the mechanisms acting to specify different cell populations. Quite paradoxically, how this pattern information is connected to cell shape remodeling for denticle formation has so far remained an unsolved question.

We show here that a single regulator, Shavenbaby, previously shown to integrate multiple patterning cascades, governs epidermal cell remodeling through the transcriptional control of several classes of effectors, acting directly in various cellular functions. This *svb*-regulated set of effectors constitutes a developmental module used in different tissues during development to produce cuticle extensions. Modification of *svb* expression has allowed the concerted evolution of this developmental module to produce morphological diversification during the evolution of insect species.

### *svb* Controls Several Aspects of Actin Organization

One of the first recognizable signs of the morphological differentiation of epidermal cells is the formation of an apical bundle of microfilaments in denticle cells [22,23]. These early steps of denticle formation depend on *svb,* which is necessary and sufficient to promote the formation of epidermal actin bundles [14]. Our results show that *svb* controls the transcription of several genes involved in different steps of actin assembly/organization. First, we found that *svb* directs the expression of *shavenoid/kojak,* a gene producing strong denticle defects when mutated and recently shown to encode a protein reported to associate with actin [32], but whose biochemical function is unknown. Second, Svb also directs the expression of *singed* and *forked,* coding respectively for the *Drosophila* putative homologs of Fascin and Espin, two proteins that crosslink parallel actin filaments and promote the formation of bundles of microfilaments [36,37]. The Forked and Singed proteins sequentially accumulate in growing denticles, a situation reminiscent of that of wing hair formation [38], suggesting that these proteins play similar roles in the formation of adult and embryonic epidermal extensions. Accordingly, the inactivation of *sn* and *f* alters denticles, strongly suggesting that denticle formation indeed involves parallel actin bundles, as shown for wing hairs.

Several cytoskeletal regulators such as dAPC, Enabled, Diaphanous/Formin, and the Arp2/3 complex, accumulate in denticles [14,23], suggesting that they are involved in denticle formation, although their respective functions remains to be evaluated. Whereas *svb* does not control the expression of those ubiquitous actin-associated factors, it is possible that *svb* regulates their activity, or subcellular localization, indirectly. Consistent with this hypothesis, we have previously shown that dAPC-2 is specifically relocalized in *svb*-induced ectopic epidermal extensions [14]. In addition, we show here that *svb* directs the epidermal expression of Wasp, a key activator of the Arp2/3 actin nucleator complex, which is well known to trigger the formation/elongation of actin filaments. Moreover, it has been shown in vitro that Fascin switches the activity of the Arp2/3 complex from the formation of a mesh-like branched network to parallel microfilaments [39], therefore suggesting that *svb* targets can regulate both the formation of actin filaments and their reorganization, at least in part, through a tight control of the activity of the Arp2/3 complex during denticle formation.

Taken together, these results show that *svb* controls the expression of several cytoskeletal factors which, probably by modifying the activity of housekeeping actin-remodeling machinery, act together to trigger the formation of apical cell extensions. Whereas few molecules are sufficient to promote actin organization in vitro, our studies indicate that, in vivo, many players are required to make a simple cellular extension. Pursuing the identification of novel genes regulated by *svb* should provide a means of identifying additional factors required for actin remodeling in vivo.

### Denticle Formation Requires a Specific Membrane/Cuticle Interaction

A surprising outcome of our studies is that denticle formation requires a specific regulation of the membrane/cuticle interaction. We show that *svb* directs the expression of *m* in trichome cells. Miniature is a single-pass membrane protein, with a short cytoplasmic tail and a large extracellular region that contains a conserved Zona Pellucida (ZP) domain [28]. ZP domains were initially identified in the three major proteins of the zona pellucida, the extracellular envelope of mammalian oocytes (see [30] for a recent review), and they are thought to be components of apical matrices [40]. We show that Miniature is required for the correct formation of denticles, revealing a novel aspect of ZP protein function in the formation of polarized cellular extensions. The absence of *m* severely impairs the interaction between the plasma membrane and cuticle layers in denticle cells, a defect likely due to a disorganization of the extracellular matrix. In the embryonic epidermis, Miniature is required for the continuous membrane/cuticle interaction that is specific to denticles, whereas only the tips of microvilli contact cuticle in naked regions [5]. The accumulation of Miniature at the base of denticles reveals the existence of a denticle-specific membrane subdomain, suggesting that additional membrane proteins might be involved in denticle formation. We found that two other ZP genes are regulated by *svb* (Table S1) and analysis of their individual role in denticle formation is under way. Our findings shed light on the importance of membrane proteins and their interaction with extracellular matrices, an aspect of cell-shape control hardly accessible to cell-culture approaches. Future analysis of Miniature targeting to the denticle should help to understand the mechanisms required for localized cell-shape modification during morphogenesis.

*svb* also regulates the expression of *y,* a gene encoding an apically secreted protein that associates with cuticle and is required for the production of black pigments [24]. While pigmentation per se is not related to cell morphogenesis, the role of Yellow in the catecholamine pathway remains elusive; it could be involved in denticle hardening, since *y* mutant larvae display defects, in the morphology of denticles, that have been proposed to account for their abnormal locomotor activity [41]. In addition, *svb* could directly regulate the local protein composition of cuticle, as suggested by the identification of an additional target encoding a putative chitin-binding protein (Table S1).

### Svb Governs a Morphological Module

**Figure 8:**
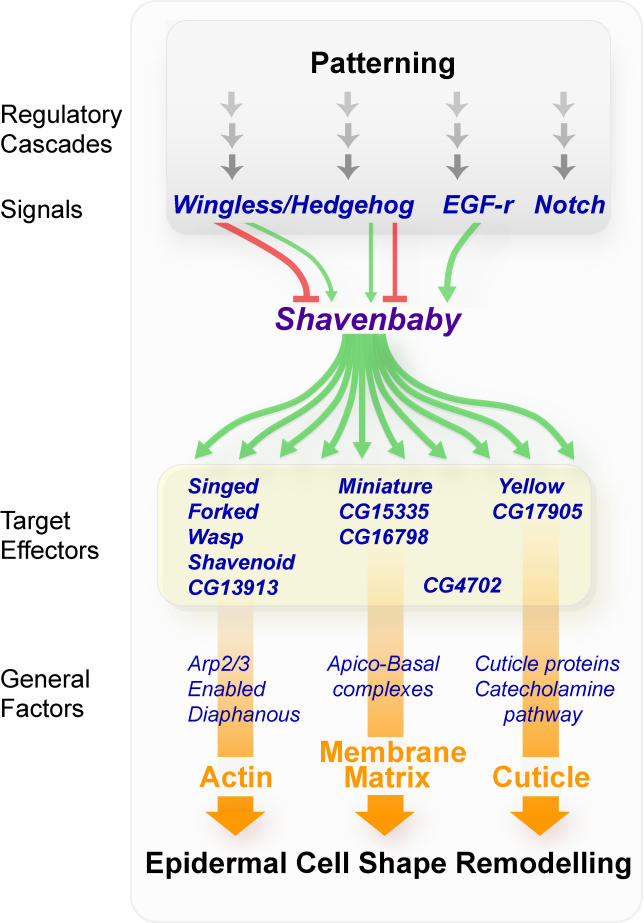
Model of *svb* Regulation and Activity during Denticle Formation During epidermal differentiation, regulatory regions governing *svb* transcription integrate outputs from many signaling pathways (Wg, Hh, and DER) and positional cues to define the precise subset of epidermal cells that express *svb*. The Shavenbaby transcription factor triggers in turn the expression of different classes of genes encoding cellular effectors. They are directly involved in distinct aspects of trichome formation, including the reorganization of actin *(singed, forked, wasp,* and *shavenoid)*, extracellular matrix *(m)* and cuticle *(y)*, likely through modifying the activity of ubiquitous cellular machineries. Additional cytoskeletal factors or regulators (independent of *svb*) might be required for the fine sculpturing of each kind of trichome, characteristic of a given body region. Modifications of *svb cis*-regulatory regions thus provide a rich source of plasticity to evolve the trichome pattern and generate morphological diversification throughout species.

Experimental evidence suggests that *svb* is situated at the bottom of regulatory cascades determining trichome patterning and is in turn directly responsible for triggering the cellular program of denticle formation (Figure 8). First, *svb* remains the most-downstream regulator determining the pattern of denticles and dorsal hairs, despite the unprecedented extent of genetic screens based upon cuticle observation (which identified most members of the Wg and DER pathways). Second, among mutations producing trichome defects, *svb* mutants display the strongest phenotype, in which most denticles and dorsal hairs are replaced by naked cuticle. Third, we show that *svb* directs the expression of genes involved in various aspects of denticle formation, including control of the cytoskeleton, membrane/matrix organization and cuticle differentiation. Finally, we provide evidence for a direct control of one of the targets *(m)*. We have defined a 400-bp *m* enhancer reproducing the endogenous expression pattern of *m* in the epidermis and show that the Svb transcription factor binds specifically to this evolutionarily conserved region. Substituting 2-bp in this *cis*-regulatory element preventing Svb binding is sufficient to abrogate its in vivo enhancer ability, thus suggesting that direct binding of Svb mediates the control of *m* epidermal expression. Several putative *svb* binding sites have been detected in evolutionarily conserved regions of other *svb* targets. Whether they are all required for *svb* transcriptional regulation remains to be tested. Further dissection of the *m* enhancer, as well as those of other *svb* targets, should lead to the definition of a functional *cis*-regulatory element responsible for *svb* control. This outcome should facilitate the identification by bioinformatic approaches of additional *svb* responsive enhancers and target genes.

We propose that *svb* directly controls the expression of a set of “effector” genes, all required for a concerted modification of cell shape and cuticle organization to achieve the formation of denticles (Figure 8). How many genes are regulated by *svb* to promote remodeling of epidermal cells? Our analysis, which covers approximately 25% of the total number of *Drosophila* genes, has led to the identification of 11 downstream targets, suggesting that *svb* activates the expression of numerous additional genes to trigger the formation of embryonic epidermal cell extensions.

Several results suggest that this *svb*-controlled module is used in different developmental programs that produce cuticle extensions. Although signaling pathways act differently in ventral and dorsal embryonic regions, we show that *svb* is also required to express the same target genes for the formation of denticles and dorsal hairs. These results show that the *svb* targets identified so far act collectively to promote the formation of various epidermal extensions, despite the fact that they display different shapes. In addition, *svb* mutations also affect the formation of adult wing hairs and antennae laterals [14], which are known to require the activity of several *svb* targets identified in the embryonic epidermis (*m, sn*, *f, sha* for adult wing hairs, and *sn, f, sha* for antennae laterals). How can the same set of *svb*-regulated effectors participate in the formation of epidermal extensions of diversified morphology? Additional cytoskeletal factors can be differentially expressed in distinct epidermal regions independently of *svb*. It is also possible that other regulators modulate the response to *svb* (cf. the weak expression of *f* and *wsp* in naked cells expressing ectopically *svb,* when compared to that of *sn, m, sha* and *y*). Finally, upstream signaling pathways certainly contribute to the sculpting of each kind of trichome through the regulation of the expression and/or activities of cellular effectors. Recent studies have shown that members of the planar polarity pathway (PCP) are indeed involved in defining denticle polarity in response to signaling pathways [23]. Interestingly, one of the identified *svb* targets, *shavenoid,* has recently been reported to interact with PCP in the adult wing, raising the possibility that such a dialog also occurs during embryonic epidermal cell remodeling. *svb* thus appears to govern a morphological module responsible for the major switch from smooth surface to trichome, the precise shape of which is finely sculptured by independent intrinsic factors and activities.

### Evolutionary Perspectives

The pattern of trichomes has been modified several times during the evolution of insects. Across the genus *Drosophila,* at least four independent evolutionary transitions have led to the loss (to various extents) of dorsal hairs. Nevertheless, evolution of the pattern of dorsal hairs and denticles results from the modification of *svb* expression in all studied cases [17–19]. Although the expression of patterning genes is unchanged in all species examined [42], the difference in the dorsal hair pattern between D. melanogaster and D. sechellia is due only to the modification of *shavenbaby*'s response to signaling pathways [16]. Our analysis further shows that the restriction of *svb* expression in D. sechellia embryos causes, in turn, the restriction of the dorsal expression of *m, sn, f, sha,* and *wsp* genes (Figure S2). Therefore, all these *svb* targets display a concerted modification of their expression in *D. sechellia,* bringing additional evidence that together they constitute a developmental module. Consistent with this interpretation, we show that individual inactivation, or experimental modifications of the expression, of any of the *svb* target genes identified so far are not sufficient to modify the trichome pattern. These data indicate that denticle formation requires multiple factors that act collectively to remodel epidermal cell shape. This requirement for many genes to build a cuticular extension doubtless constitutes a developmental constraint, explaining why modifications of the expression of *svb,* the factor that governs this entire set of genes, are required for the trichome pattern to evolve.

Evolutionary modifications of *cis*-regulatory elements of *lin-48,* the putative *svb* homolog in worms, were responsible for the difference in the position of the excretory duct between Caenorhabditis elegans and C. briggsae [43]. Although accumulated data thus demonstrate the particular role of *svb* genes in morphological diversification between relatively close species, how they are related to the evolution of animal forms across more distant phyla remains an open question. Several features of *svb* function have been conserved in mammals, including the role of one of its homologs, *m-ovo1,* in the differentiation of epidermal derivatives [44] and its regulation by the Wnt pathway [45]. Our identification of genes regulated by *svb* in flies now opens the way to evaluate the contribution of the different parameters contributing to the role of *svb* in morphological evolution, from the modification of its response to signaling pathways to that of its cellular targets.

## Materials and Methods

### Fly strains and transgenic constructs.

We used *UAS-svb; UAS-ovoA; Ptc-Gal4; Wg-Gal4; svb^1^; btd^1^, svb^1^; svbR9; w,m^1^; sn^3^; f^3^; f^5^; f^36a^; sha^1^; sha^V15^; sn^3^,f^36a^; m^1^,f^36a^; m^1^,sn^3^; m^1^,sn^3^, f^36a^; wsp^1^; wsp^3^; m^1^,sn^3^, f^36a^; m^1^,sn^3^, f^36a^;sha^1^; m^1^,sn^3^,f^36a^;sha^V15^; m^1^,sn^3^, f^36a^;wsp^3^; w, Df(1)m-MR; P(EP)EP345,* and a *w D. sechellia* stock from the D. Stern Laboratory. Stocks with multiple mutations were obtained by recombination, and mutant chromosomes were kept over balancers carrying Kr-Gal4>>UAS-GFP transgenes [46]. Transgenic lines were generated using standard P-element transformation. Based on the 2003 genome release, the 6kmin construct corresponds to genomic position chrX:11585555–11591094 and 11596605–11597139, 3kmin to chrX:11585555–11588410; positions, both inserted into pCasPER β-gal. Wild-type, or site-directed mutagenized, 0.4Kmin enhancer was amplified from genomic regions (11487355–11487753) and cloned into the pCβ vector (carrying a nuclear-LacZ reporter) and in the pGal4 vector for rescue experiments. A full-length *m* cDNA inserted into the pUASt vector was used to generate pUAS-min transgenic lines. Details of cloning strategy are available upon request.

### Embryo staining.

Antisense probes derived from cDNAs or genomic fragments were synthesized in vitro and processed for in situ hybridization following standard procedures. To identify *svb* mutant embryos, we used either a *svb* mutant chromosome carrying the *btd1* mutation (leading to head defects; a gift from E. Wieschaus), or embryos sorted using the GFP balancer. For high-resolution imaging, ventral epidermis was hand-dissected. Immunostaining was performed according to [14], with anti-Miniature [28] at 1/400, anti-Forked [37] at 1/300, anti-Singed (Development Studies Hybridoma Bank, Iowa City, Iowa, United States) at 1/2, anti β-galactosidase (Cappel, Solon, Ohio, United States), Alexafluor488-labeled antibodies (Molecular Probes, Eugene, Oregon, United States) and TRITC-phalloidin (Sigma, St. Louis, Missouri, United States). Embryos were mounted in Vectashield (Vector Laboratories, Burlingame, California, United States) and photographed with a Leica TSP2 confocal microscope (Wetzlar, Germany).

### Cuticle preparation and electronic microscopy.

Live mutant embryos (lacking GFP fluorescence from balancers) were hand-selected and cuticles prepared in hoyers/lactic acid (1/1). Abdominal segments 3 and 4 were imaged using a Zeiss Axioplan microscope equipped with phase contrast (Zeiss, Oberkochen, Germany). All high-magnification pictures correspond to the midline region of the fourth abdominal segment. For scanning electron microscopy, stage 16 embryos were fixed 15 min in heptane saturated in glutaraldehyde, devitellinized by hand and, after several water washes, dehydrated through an ethanol series. They were then dried using CO_2_ in a BOMAR SPC-900/EX critical-point dryer (Bomar, Tacoma, Washington, United States), sputtered (MED 020, Bal-Tek, Balzers, Liechtenstein) with a 20-nm gold palladium coat and examined with a Philips XL30 field emission microscope. For transmission electron microscopy, stage 16 embryos, fixed and devitellinized as above, were post-fixed for 1 h in para-formaldehyde 3%, glutaraldehyde 0.5% in PBS and then in 1% osmium-tetroxide for 1h. After dehydration, samples were embedded in Epon. Sections were stained with uranyl acetate and photographed with a Leo 510 (Zeiss) microscope.

### Electrophoretic mobility shift assay and in vitro DNA-binding.

DNA binding assays were performed as previously described [29]. Wild-type or site-directed mutagenized *m* DNA probes (position 11487606–11487768) were synthesized by PCR, end-labeled and incubated with recombinant proteins, produced in bacteria, and purified according to the manufacturer's specifications (Pharmacia, Uppsala, Sweden). We used GST-SVB, a fusion protein with the 450 C-terminal aa of Svb encompassing the DNA binding domain, and GST alone as control. Electrophoresis gels were analyzed using a Bio imaging analyzer (Fuji, Tokyo, Japan).

## Supporting Information

Figure S1 Time-Course Analysis of the Distribution of Forked, Singed, and Miniature Proteins during Remodeling of Ventral Epidermal Cells Embryos were collected for 1 h at 18 °C, then allowed to develop at 25 °C for a period corresponding to 11, 12, and 14 h, before being processed for actin and immunological staining. Confocal views of abdominal segments A3 and A4 were recorded using a Leica TSP 2 microscope.
(2.6 MB JPG)

Figure S2 Evolution of *svb* Target Gene Expression in D. sechellia Embryos Epidermal expression of *wasp, forked, singed,* and *shavenoid* in stage 15 embryos from D. melanogaster (left) and D. sechellia (right) species. All pictures are lateral views, corresponding to the same abdominal segments. Anterior is to the left and dorsal is up.
(513 KB JPG)

Table S1 Summary of Candidate Genes Tested for Their Putative Regulation by the Shavenbaby Transcription Factor Candidates were selected by three independent criteria. (1) Expression pattern: we searched for genes transcribed in a subset of epidermal cells, by conducting a survey of the project of systematic determination of patterns of gene expression in *Drosophila* embryogenesis that runs in the Berkeley Drosophila Genome Project (BDGP;  http://toy.lbl.gov:8888/cgi-bin/ex/insitu.pl), and from published literature. (2) Denticle phenotype: we selected genes reported to specifically affect denticle formation when mutated. (3) Putative function: we selected candidates known to be involved in a biological process that is implicated in denticle formation, either actin remodeling, cuticle pigmentation, or cuticle formation.
We confirmed or established the expression pattern of candidates by in situ hybridization, and determined whether their expression depended upon *svb* activity by comparing their expression between wild-type and *svb* mutant embryos. Each candidate showing a strong reduction of its epidermal expression in *svb* mutants was further validated through the analysis of consequences both of *svb* ectopic expression *(wg≫svb)* and of the inhibition of *svb* activity in a subset of denticle cells *(ptc≫OvoA)*. The different columns refer to the name of candidates, their synonym, their respective Flybase identifier (http://flybase.bio.indiana.edu/), their putative function, a summary of their embryonic expression at the time of epidermal differentiation and their dependence on *svb* activity.
CNS, central nervous system; Ep, epidermal; NT, not tested.
(35 KB PDF)

Table S2 Quantification of Denticle Defects Resulting from Inactivation of *svb* Downstream Targets The denticle belt of the fourth abdominal segment of wild-type and simple or multiple mutant embryos was photographed at high resolution (using a 63× phase contrast objective, NA [numerical aperture] = 1.4, and a digital Nikon CCD camera). Pictures were processed and analyzed using the ImageJ software (http://rsb.info.nih.gov/ij). We focused on the fourth row of denticles, which are relatively large and display a characteristic stereotyped morphology. Analyses were performed on five to ten individuals of the same genotype, to obtain a number of denticles superior to 70.
(A) Summary of the consequences of *svb* target inactivation on denticle formation. We measured several geometrical parameters, including denticle area, perimeter, width, and height. We also determined the number of denticles that are present along the fourth row (*, we focused on a 75 μm-wide region, corresponding to the most ventral part). Statistical analyses (3 way ANOVA, with denticle being nested in individuals, which is nested in genotype on a given variable) confirm that despite intragroup variations, differences between genotypes can be considered as highly significant (*p* < 0.001). The only exception is between *m1* and *Df(1)mr*, which is not statistically different, in a good agreement with the fact the *m1* mutation is reported to be a null *m* allele.
(B) Histograms plot the respective effects of *svb* targets on denticle size (as estimated by the area) and number along the row. Inactivation of each individual target decreases denticle size to various extents, a phenotype aggravated when mutations are cumulated. The absence of *shavenoid* causes a severe reduction of the size resulting from multiple split denticles, as indicated by the increase of the apparent denticle number (and confirmed by scanning electron microscopy analyses). Combination of *m* and *sn* (and/or *f*) also leads to denticle splitting, albeit at a lesser extent. The simultaneous inactivation of *m, sn, f,* and *sha* results in a strong reduction of the number of denticles that are replaced by naked regions, indicating a functional interaction between these genes for denticle formation.
(1.1 MB JPG)

## Abbreviations

bp: base pair
kb: kilobase
0.4KminKO: 0.4Kmin knockout
