# Supplementary material for: Shavenbaby Couples Patterning to Epidermal Cell Shape Control
Source: PLoS Biol. 2006 Aug 22;4(9):e290. doi: 10.1371/journal.pbio.0040290 (PMC1551925; doi:10.1371/journal.pbio.0040290)
Supplement: Supplementary file 1 [file pbio.0040290.st001.pdf]

Expression pattern

name	*synonym*	*FlyBase ID*	*function*	*expression*	*svb*	*Ref.*
CG11380	EG:BACR42I17.7	FBgn0040359	unknown	Ep stripes	no	[1, 2]
**CG13913**		**FBgn0035190**	**actin binding**	**Ep stripes**	**yes**	**[2, 3]**
CG14626	EG:BACR42I17.6	FBgn0040360	unknown	Ep stripes	no	[1, 2]
CG14643		FBgn0037225	unknown	Ep stripes	no	[1, 2]
CG14960		FBgn0035428	unknown	Ep stripes	no	[1, 2]
CG15282	DS07721.3<br>BG:DS07721.3<br>BcDNA:RE15191	FBgn0028855	unknown	Ep stripes	no	[1, 2]
**CG15335**	**CG15338**	**FBgn0030001**	**ZP**	**Ep stripes**	**yes**	**[1, 2]**
CG15589		FBgn0037409	unknown	Ep stripes	no	[1, 2]
**CG16798**	**38C.41**	**FBgn0032856**	**ZP-like**	**Ep stripes**	**yes**	[1, 2]
CG16885	CG18634	FBgn0032538	electron transport	Ep stripes	no	[1, 2]
**CG17905**		**FBgn0032598**	**chitin binding**	**Ep stripes**	**yes**	[1, 2]
CG2467		FBgn0030355	ZP-like	Ep stripes	no	[1, 2]
CG31559	CG11461, CG15584	FBgn0051559	electron transport	Ep stripes	no	[1, 2]
CG32159	CG13034, CG4541	FBgn0052159	unknown	Ep stripes	no	[1, 2]
CG3244		FBgn0031629	sugar binding	Ep stripes	no	[1, 2]
CG4115		FBgn0038017	sugar binding	Ep stripes	no	[1, 2]
**CG4702**		**FBgn0037992**	**unknown**	**Ep stripes**	**yes**	[1, 2]
CG7356		FBgn0031975	acyltransferase activity	Ep stripes	no	[1, 2]
CG9460		FBgn0033115	serine-type endopeptidase inhibitor activity	Ep stripes	no	[1, 2]
CG9517		FBgn0030591	electron transport	Ep stripes	no	[1, 2]
CG9689	BcDNA:GH05731	FBgn0030159	unknown	Ep stripes	no	[1, 2]
dumpy	CG33196<br>BcDNA:SD02173	FBgn0053196	ZP	Ep stripes	no	[2, 4]
four-jointed	CG10917	FBgn0000658	Membrane protein	Ep stripes	no	[1, 2]
fringe	CG10580	FBgn0011591	acetyl-glucosamine transferase	Ep stripes	no	[1, 2]
GalNAc-T1	CG8182	FBgn0034025	N-acetylgalactosaminyltransferase	Ep stripes	no	[1, 2]
Hsp68	CG5436	FBgn0001230	ATP binding, Heat shock protein	Ep stripes	no	[1, 2]
ken	CG5575	FBgn0011236	Transcription factor	Ep stripes	no	[1, 2]

**miniature**	**CG9369**	**FBgn0002577**	**ZP**	**Ep stripes**	**yes**	**[2, 5]**
ogre	CG3039	FBgn0004646	Innexin, gap junction	Ep stripes	no	[1, 2]
osiris17	CG15598	FBgn0037427	unknown	Ep stripes	no	[1, 2]
osiris6	CG1151	FBgn0027527	unknown	Ep stripes	no	[1, 2]
pale	CG10118	FBgn0005626	tyrosine 3-monooxygenase	Ep stripes	no	[1, 2]
piopio	CG3541	FBgn0020521	ZP	Ep stripes	no	[2, 6]
quail	CG6433	FBgn0003187	actin binding	Ep stripes	no	[1, 2]
rps8	CG7808	FBgn0039713	protein synthesis	Ep stripes	no	[1, 2]
**singed**	**CG32858**	**FBgn0003447**	**actin binding**	**Ep stripes**	**yes**	[1, 2]
**wasp**	**CG1520**	**FBgn0024273**	**actin binding**	**Ep stripes**	**yes**	[1, 2]

Denticle phenotype

name	*synonym*	*Flybase ID*	*function*	*expression*	*svb*	*Ref.*
**forked**	**CG5424**	**FBgn0000630**	**actin binding**	**Ep stripes**	**yes**	**[2, 7]**
fritz	CG17657	FBgn0020302	establishment of planar polarity; wing hair site selection	ubiquitous	NT	[2, 8]
Hem	Kette, CG5837	FBgn0011771	Arp2/3 regulator	ubiquitous,CNS	NT	[9]
**shavenoid**	**kojak, CG13209**	**FBgn0003382**	**actin binding**	**Ep stripes**	**yes**	**[2, 10, 11]**
**singed**	**CG32858**	**FBgn0003447**	**actin binding**	**Ep stripes**	**yes**	**[2, 7]**
stubble	CG4316	FBgn0003319	peptidase	undetectable	no	[2, 7]
tricornered	CG8637	FBgn0003744	DBF2 kinase	ubiquitous	NT	[2, 12]

Putative function

name	synonym	Flybase ID	function	expression	svb	Ref.
**1) Actin remodelling**						
ArcP34	CG10954	FBgn0032859	Arp2/3 complex (ARPC2)	ubiquitous	NT	[1, 2]
Arp87C	CG6174	FBgn0011745	actin binding (Arp1)	ubiquitous	NT	[1]
chickadee	CG9553	FBgn0000308	Profilin	ubiquitous	no	[2]
dAPC2	CG6193	FBgn0026598	Wg signalling, cytoskeleton	ubiquitous	NT	[13]
diaphanous	CG1768	FBgn0011202	FH domain	weak ubiquitous, gut	no	[2, 14]
enabled	CG15112	FBgn0000578	ENA/VASP	weak ubiquitous, CNS	no	[2, 14]
fimbrin	CG8649	FBgn0024238	actin binding	ubiquitous, CNS	no	[2]
**forked**	**CG5424**	**FBgn0000630**	**actin binding**	**Ep stripes**	**yes**	**[2, 7]**
gelsolin	CG1106	FBgn0010225	actin binding	weak ubiquitous	no	[2]
**singed**	**CG32858**	**FBgn0003447**	**actin binding**	**Ep stripes**	**yes**	**[2, 7]**
sop2	CG8978	FBgn0001961	Arp2/3 complex (ARPC1)	ubiquitous, muscle	no	[2]
twinstar	CG4254	FBgn0011726	cofilin, ADF	ubiquitous	no	[2]
**wasp**	**CG1520**	**FBgn0024273**	**actin binding**	**Ep stripes**	**yes**	[1, 2]
**2) Pigmentation**						
amd	CG10501	FBgn0000075	aromatic Laa decarboxylase	Ep stripes	no	[2, 15]
Black cells	CG5779	FBgn0000165	monophenol oxydase	crystal cells	no	[1, 2]
bursicon	CG13419	FBgn0038901	neuropeptide Hormone	neurons	NT	[16]
CG8193	CG8193	FBgn0033367	monophenol oxydase	crystal cells	no	[2, 17]
DAT	CG3318	FBgn0019643	dopamine N-acetyltransferase	no epidermis, oenocytes and other	no	[2, 18]
ddc	CG10697	FBgn0000422	dopa decarboxylase	Ep stripes	no	[2, 10, 19]
Dox-A2	CG10484	FBgn0000486	diphenol oxydase, proteasome	ubiquitous	no	[2]
Dox-A3	CG2952	FBgn0000487	diphenol oxydase	crystal cells	no	[2, 17]
ebony	CG3331	FBgn0000527	b-alanyl dopamine synthase	oenocytes	no	[2, 20]
pale	CG10118	FBgn0005626	tyrosine hydroxylase	Ep stripes	no	[2, 21, 22]

silver	CG18503	FBgn0004648	carboxypeptidase	hemocytes, CNS, anal pads	NT	[23]
tan	CG6586	FBgn0028980	b-alanyl dopamine hydrolase	***wrong annotation!***	NT	
punch	unpigmented, CG9441	FBgn0003162	GTP cyclohydrolase, TH regulator	CNS	NT	[10, 24]
**yellow**	**CG3757**	**FBgn0004034**	**catecholamine**	**Ep stripes**	**yes**	**[2, 25]**
faintoid	quicksilver	FBgn0003186	*uncloned*		NT	[26]
faint		FBgn0000631	*uncloned*		NT	[10]

3) General cuticle defects

disembodied	CG12028	FBgn0000449	Ecdysone synthesis (22-hydroxylase)	ring-gland	NT	[21, 27]
grainy head	CG5058	FBgn0004586	TF	ubiquitous in epidermis	NT	[10, 28]
krotzkopf verkehrt	CG2666	FBgn0001311	chitin synthase	trachea, weak epidermis	NT	[29]
knickkopf	CG6217	FBgn0001321	dopamine beta-monooxygenase	trachae, weak epidermis	NT	[29]
mummy	CG9535	FBgn0027501	UDP-Nacetylglucosamine diphosphorylase	weak epidermis	NT	[10, 30, 31]
phantom	CG6578	FBgn0004959	Ecdysone synthesis (25-hydroxylase)	ring-gland	NT	[32]
pimples	CG5052	FBgn0003087	separase	brain, not epidermal	NT	[10, 33]
sec-61 beta	CG10130	FBgn0010638	exocyst	ubiquitous	NT	[34]
shade	CG13478	FBgn0003388	Ecdysone synthesis (20-hydroxylase)	weak epidermis	NT	[21, 35]
shadow	CG14728	FBgn0003312	Ecdysone synthesis (2-hydroxylase)	ring-gland	NT	[21, 36]
shroud	Kayak ; CG15509	FBgn0001297	D-Fos	amnioserosa, gut	NT	[21, 37, 38]
spook	CG10594	FBgn0003486	Ecdysone synthesis	amnioserosa	NT	[21]
syntaxin 1A	CG5448	FBgn0013343	t-SNARE	ubiquitous	NT	[39]
Pipsqueak	CG2368, zeppelin	FBgn0004399	Transcription Factor		NT	[29]
filzig		FBgn0000712	*uncloned*		NT	[10]
ghost		FBgn0001106	*uncloned*		NT	[21]
haunted		FBgn0001178	*uncloned*		NT	[21]

References

1. Tomancak, P., et al., *Systematic determination of patterns of gene expression during Drosophila embryogenesis.* Genome Biol, 2002. **3**(12): p. RESEARCH0088.
2. This_study.
3. Staudt, N., et al., *Gain-of-function screen for genes that affect Drosophila muscle pattern formation.* PLoS Genet, 2005. **1**(4): p. e55.
4. Wilkin, M.B., et al., *Drosophila dumpy is a gigantic extracellular protein required to maintain tension at epidermal-cuticle attachment sites.* Curr Biol, 2000. **10**(10): p. 559-67.
5. Roch, F., C.R. Alonso, and M. Akam, *Drosophila miniature and dusky encode ZP proteins required for cytoskeletal reorganisation during wing morphogenesis.* J Cell Sci, 2003. **116**(Pt 7): p. 1199-207.
6. Jazwinska, A., C. Ribeiro, and M. Affolter, *Epithelial tube morphogenesis during Drosophila tracheal development requires Piopio, a luminal ZP protein.* Nat Cell Biol, 2003. **5**(10): p. 895-901.
7. Dickinson, W.J. and J.W. Thatcher, *Morphogenesis of denticles and hairs in Drosophila embryos: involvement of actin-associated proteins that also affect adult structures.* Cell Motil Cytoskeleton, 1997. **38**(1): p. 9-21.
8. Collier, S., et al., *The WD40 repeat protein fritz links cytoskeletal planar polarity to frizzled subcellular localization in the Drosophila epidermis.* Genetics, 2005. **169**(4): p. 2035-45.
9. Hummel, T., K. Leifker, and C. Klambt, *The Drosophila HEM-2/NAP1 homolog KETTE controls axonal pathfinding and cytoskeletal organization.* Genes Dev, 2000. **14**(7): p. 863-73.
10. Nusslein-Volhard, C., E. Wieschaus, and H. Kluding, *Mutations affecting the pattern of the larval cuticle in Drosophila melanogaster.* Roux Arch. Dev. Biol., 1984. **193**: p. 267-282.
11. Ren, N., et al., *The shavenoid Gene of Drosophila Encodes a Novel Actin Cytoskeleton Interacting Protein That Promotes Wing Hair Morphogenesis.* Genetics, 2006. **172**(3): p. 1643-53.
12. Geng, W., et al., *The tricornered gene, which is required for the integrity of epidermal cell extensions, encodes the Drosophila nuclear DBF2-related kinase.* Genetics, 2000. **156**(4): p. 1817-28.
13. Akong, K., et al., *Drosophila APC2 and APC1 play overlapping roles in wingless signaling in the embryo and imaginal discs.* Dev Biol, 2002. **250**(1): p. 91-100.
14. Price, M.H., et al., *Cytoskeletal dynamics and cell signaling during planar polarity establishment in the Drosophila embryonic denticle.* J Cell Sci, 2006. **119**(Pt 3): p. 403-15.
15. Marsh, J.L., M.P. Erfle, and C.A. Leeds, *Molecular localization, developmental expression and nucleotide sequence of the alpha-methyldopa hypersensitive gene of Drosophila.* Genetics, 1986. **114**(2): p. 453-67.
16. Dewey, E.M., et al., *Identification of the gene encoding bursicon, an insect neuropeptide responsible for cuticle sclerotization and wing spreading.* Curr Biol, 2004. **14**(13): p. 1208-13.
17. Waltzer, L., et al., *Two isoforms of Serpent containing either one or two GATA zinc fingers have different roles in Drosophila haematopoiesis.* Embo J, 2002. **21**(20): p. 5477-86.
18. Hintermann, E., et al., *Cloning of an arylalkylamine N-acetyltransferase (aaNAT1) from Drosophila melanogaster expressed in the nervous system and the gut.* Proc Natl Acad Sci U S A, 1996. **93**(22): p. 12315-20.
19. Hirsh, J., B.A. Morgan, and S.B. Scholnick, *Delimiting regulatory sequences of the Drosophila melanogaster Ddc gene.* Mol Cell Biol, 1986. **6**(12): p. 4548-57.
20. Hovemann, B.T., et al., *The Drosophila ebony gene is closely related to microbial peptide synthetases and shows specific cuticle and nervous system expression.* Gene, 1998. **221**(1): p. 1-9.
21. Jurgens, G., et al., *Mutations affecting the pattern of the larval cuticle in Drosophila melanogaster.* Roux Arch. Dev. Biol., 1984. **193**: p. 283-295.
22. Neckameyer, W.S. and W.G. Quinn, *Isolation and characterization of the gene for Drosophila tyrosine hydroxylase.* Neuron, 1989. **2**(2): p. 1167-75.
23. Bourbon, H.M., et al., *A P-insertion screen identifying novel X-linked essential genes in Drosophila.* Mech Dev, 2002. **110**(1-2): p. 71-83.
24. McLean, J.R., S. Krishnakumar, and J.M. O'Donnell, *Multiple mRNAs from the Punch locus of Drosophila melanogaster encode isoforms of GTP cyclohydrolase I with distinct N-terminal domains.* J Biol Chem, 1993. **268**(36): p. 27191-7.
25. Geyer, P.K. and V.G. Corces, *Separate regulatory elements are responsible for the complex pattern of tissue-specific and developmental transcription of the yellow locus in Drosophila melanogaster.* Genes Dev, 1987. **1**(9): p. 996-1004.
26. Wieschaus, E., C. Nusslein-Volhard, and G. Jurgens, *Mutations affecting the pattern of the larval cuticle in Drosophila melanogaster.* Roux Arch. Dev. Biol., 1984. **193**: p. 296--307.
27. Chavez, V.M., et al., *The Drosophila disembodied gene controls late embryonic morphogenesis and codes for a cytochrome P450 enzyme that regulates embryonic ecdysone levels.* Development, 2000. **127**(19): p. 4115-26.
28. Bray, S.J., et al., *Embryonic expression pattern of a family of Drosophila proteins that interact with a central nervous system regulatory element.* Genes Dev, 1989. **3**(8): p. 1130-45.
29. Ostrowski, S., H.A. Dierick, and A. Bejsovec, *Genetic control of cuticle formation during embryonic development of Drosophila melanogaster.* Genetics, 2002. **161**(1): p. 171-82.
30. Araujo, S.J., et al., *mummy/cystic encodes an enzyme required for chitin and glycan synthesis, involved in trachea, embryonic cuticle and CNS development--analysis of its role in Drosophila tracheal morphogenesis.* Dev Biol, 2005. **288**(1): p. 179-93.
31. Tonning, A., et al., *Hormonal regulation of mummy is needed for apical extracellular matrix formation and epithelial morphogenesis in Drosophila.* Development, 2006. **133**(2): p. 331-41.
32. Warren, J.T., et al., *Phantom encodes the 25-hydroxylase of Drosophila melanogaster and Bombyx mori: a P450 enzyme critical in ecdysone biosynthesis.* Insect Biochem Mol Biol, 2004. **34**(9): p. 991-1010.
33. Stratmann, R. and C.F. Lehner, *Separation of sister chromatids in mitosis requires the Drosophila pimples product, a protein degraded after the metaphase/anaphase transition.* Cell, 1996. **84**(1): p. 25-35.
34. Valcarcel, R., et al., *Sec61beta, a subunit of the protein translocation channel, is required during Drosophila development.* J Cell Sci, 1999. **112 (Pt 23)**: p. 4389-96.
35. Petryk, A., et al., *Shade is the Drosophila P450 enzyme that mediates the hydroxylation of ecdysone to the steroid insect molting hormone 20-hydroxyecdysone.* Proc Natl Acad Sci U S A, 2003. **100**(24): p. 13773-8.
36. Warren, J.T., et al., *Molecular and biochemical characterization of two P450 enzymes in the ecdysteroidogenic pathway of Drosophila melanogaster.* Proc Natl Acad Sci U S A, 2002. **99**(17): p. 11043-8.
37. Giesen, K., et al., *Regulation of glial cell number and differentiation by ecdysone and Fos signaling.* Mech Dev, 2003. **120**(4): p. 401-13.
38. Namiki, T., et al., *Cytochrome P450 CYP307A1/Spook: a regulator for ecdysone synthesis in insects.* Biochem Biophys Res Commun, 2005. **337**(1): p. 367-74.
39. Wu, M.N., et al., *Syntaxin 1A interacts with multiple exocytic proteins to regulate neurotransmitter release in vivo.* Neuron, 1999. **23**(3): p. 593-605.
